# The effect of systematic couple group therapy on families with depressed juveniles: a pilot trial

**DOI:** 10.3389/fpsyt.2024.1283519

**Published:** 2024-05-28

**Authors:** Tian-Jiao Meng, Ying Qian, Yu-Lu Wang, Bing-Ling Gao, Jia-Jia Liu, Jing-Li Yue, Deng-Hua Tang

**Affiliations:** ^1^ Peking University Sixth Hospital, Peking University Institute of Mental Health, NHC Key Laboratory of Mental Health (Peking University), National Clinical Research Center for Mental Disorders (Peking University Sixth Hospital), Beijing, China; ^2^ School of Nursing, Peking University, Beijing, China

**Keywords:** adolescent, communication, couples therapy, depression, family conflict

## Abstract

**Background:**

Depression is a primary cause of illness and disability among teenagers, and the incidence of depression and the number of untreated young people have increased in recent years. Effective intervention for those youths could decrease the disease burden and suicide or self-harm risk during preadolescence and adolescence.

**Objective:**

To verify the short efficacy of the systemic couple group therapy (SCGT) on youths’ depression changes and families with depressed adolescents.

**Methods:**

The study was a self-control trial; only within-group changes were evaluated. Participants were couples with a depressed child who was resistant to psychotherapy; they were recruited non-randomly through convenient sampling. The paired-sample *t*-test and Wilcoxon signed-rank test were used to compare differences before and after interventions. The effect sizes were also estimated using Cohen’s d. Spearman’s correlation analysis was used to examine associations between changes.

**Results:**

A downward trend was seen in depressive symptoms after treatment, and Cohen’s d was 0.33 (*p* = 0.258). The adolescents perceived fewer interparental conflicts, and the effect sizes were medium for perceived conflict frequency (0.66, *p* = 0.043), conflict intensity (0.73, *p* = 0.028), conflict solutions (0.75, *p* = 0.025), coping efficacy (0.68, *p* = 0.038), and perceived threat (0.57, *p* = 0.072). For parents, global communication quality, constructive communication patterns, and subjective marital satisfaction significantly improved after interventions, with large effect sizes (1.11, 0.85, and 1.03, respectively; all *p <* 0.001). Other destructive communication patterns such as demand/withdraw (*p* = 0.003) and mutual avoidance (*p* = 0.018) and communication strategies like verbal aggression (*p* = 0.012), stonewalling (*p* = 0.002), avoidance–capitulation (*p* = 0.036), and child involvement (*p* = 0.001) also reduced, with medium effect sizes (0.69, 0.52, 0.55, 0.71, 0.46, and 0.79, respectively). Meanwhile, the associations between depression changes and changes in interparental conflicts (*p <* 0.001) and marital satisfaction (*p* = 0.001) were significant.

**Conclusions and clinical relevance:**

The SCGT offers the possibility for the treatment of families with depressed children who are unwilling to seek treatment. Helping parents improve communication and marital quality may have benefits on children’s depressive symptoms.

## Introduction

Depression is a serious mental health problem during adolescence; the prevalence of the youth population experiencing depression at any one time is 2.6% (1, 2). Depression is a leading cause of youth illness and disability and also predicts a wide range of long-term negative effects, including mental disorders in adulthood, educational under-achievement, and increased risk of self-harm and suicidal behavior (3). The prevalence of depression and the number of young people with untreated depression have increased in recent years (4). Longer duration of untreated depression leads to greater severity, poorer prognosis, higher suicide and self-harm risk, and cognitive impairment (5). Effective intervention for those youths with depression could decrease the disease burden and suicide and self-harm risk during preadolescence and adolescence, a sensitive period of social and neural development (6).

The current National Institute for Health and Care Excellence (NICE) guidelines for the treatment of moderate/severe depression in youths recommend individual cognitive behavioral therapy (CBT), interpersonal therapy (IPT), family therapy, or psychodynamic psychotherapy for at least 3 months (7). However, there are also difficulties in engaging depressed youths in psychotherapy. Recent trials have demonstrated a significant level of drop-out from CBT and other therapies (8). Children are usually embedded in a family context and dependent on their parents for nurturance, support, and assistance; depression does, of course, run in families (9).

Findings point to the negative impacts of exposure to high interparental conflicts on youth’s adjustment problems, aggression, conduct disorders, anxiety, and depression (10, 11). The interparental conflicts influence the family environment in which children learn and grow. Children are also likely exposed to indirect effects of interparental conflicts, which affect parenting behaviors—the effects “spilling over” from the interparental relationship to the parent–child relationship (12). Partners who are satisfied with and receive support from their spouse tend to be more available and responsive to their children’s needs and vice versa (13). High marital conflicts, low marital satisfaction and fitness, and maladaptive communication and problem-solving are risk factors associated with depression in children (14, 15). For depressed youths with difficulties engaging in psychotherapy, parental involvement in the therapy may help reduce those risk factors, bringing direct or indirect benefits for depressed children.

In the present study, we employed systemic couple group therapy (SCGT) focusing on interparental conflicts to verify 1) the efficacy on marital quality and 2) the good impact on children. We included the listed variables for family, e.g., interparental communication satisfaction, marital satisfaction and fitness, and adolescents’ perception of interparental conflicts and interparental communication, which have been proven to impact depressive symptoms of adolescents (6 10). We postulated that interparental communication, marital satisfaction, and fitness will be improved and that perceived interparental conflicts and depression in children will change along with the change in interparental relationships. This study could offer a potential approach to intervention in depressed youths unwilling to participate in therapy.

## Methods

The design of the present study was a self-control trial. We focused on the within-group effects before and after the SCGT intervention for parents with depressed youths. The study was approved by the Biomedical Ethics Committee of Peking University Sixth Hospital. The findings of the study were reported according to the Strengthening the Reporting of Observational Studies in Epidemiology (STROBE) checklist.

### Participants

Couples were eligible for the study if 1) their current marital status ≥1 year; 2) their dyadic adjustment scale (DAS) ≤95 reported by at least one spouse; 3) they are Chinese; 4) one of their children is diagnosed with depressive disorders according to the International Classification of Diseases (ICD), 11th edition, without medication or not under stable medication during the treatment period; 5) their children <18 years; 6) their depressed adolescents exhibit resistance to participating in psychotherapy; and 7) their children stay in contact with one or both parents. Exclusion criteria were as follows: 1) other urgent issues, i.e., active suicidal intent, serious self-harm, and frequent non-attendance; 2) other comorbid disorders required exclusion such as schizophrenia and substance addiction.

Participants were recruited through convenient sampling in mainland China from October 2021 to January 2022. Written informed consent was obtained from all participants.

### Measures

Demographic characteristics included age, education, family income, and marriage length. The dyadic adjustment scale (DAS), quality of marriage index (QMI), conflicts and problem-solving scale (CPS), communication patterns questionnaire (CPQ), and primary communication inventory (PCI) were used in the present study.

The DAS is a 32-item inventory scored on a Likert scale, which can reflect marriage adjustment and satisfaction among couples (16). It contains four dimensions: affection expression, dyadic consensus, dyadic cohesion, and marital satisfaction. Higher scale scores represent greater marital satisfaction. The cutoff score of the DAS is 107, which is considered an indicator of serious distress in married couples (17). In the present study, Cronbach’s α values were 0.95 and 0.96 for husband and wife, respectively.

The perceived quality of marital relationship (marital satisfaction) was assessed using the QMI. The QMI is a six-item scale that asks spouses to rate on a 9-point scale (18). The higher the score (ranging from 6 to 45), the better the marital quality. In the present study, Cronbach’s α values were 0.98 and 0.98 for husband and wife, respectively.

The CPS was utilized to measure specific marital conflict strategies. The CPS is a 44-item questionnaire that comprises four conflict dimensions (i.e., frequency, severity, resolution, and efficacy) and six conflict strategy subscales (i.e., cooperation, avoidance–capitulation, stonewalling, verbal aggression, physical aggression, and child involvement) (19). In the present study, Cronbach’s α values were 0.76–0.95 and 0.69–0.95 for husband and wife, respectively, on each dimension.

The CPQ is designed to gauge the extent to which couples employ conflict patterns when dealing with relationship problems. Each partner indicates what typically occurs in their relationship on a 9-point scale. The CPQ measures mutual discussion, understanding, and problem-solving (mutual constructive communication), and demand/withdraw (demand/withdraw communication) (20).

The PCI adopted here is a 19-item 5-point Likert scale that assesses verbal and non-verbal communication between partners (21). Higher scores reflect greater satisfaction with a couple’s communication level (22). In the present study, Cronbach’s α values were 0.94 and 0.92 for husband and wife, respectively. Communication frequency in the last month was assessed using a 5-point scale adapted from the communication scale of the partnership questionnaire. A higher score means more communication frequencies.

The children’s perception of interparental conflict scale (CPIC) was used to examine the relationship between interparental conflict, and child behavioral and emotional problems (23, 24). The CPIC has four subscales: conflict properties (i.e., conflict frequency and intensity), triangulation/stability (i.e., enduring aspects of conflict as well as the degree to which children feel caught between parents), self-blame (i.e., the extent to which children blame themselves for interparental conflict), and perceived threat regarding potential negative consequences of interparental conflict, such as divorce.

Children’s depression was assessed using the children’s depression inventory (CDI). The CDI has five subscales: anhedonia, negative affect, low self-esteem, low efficacy, and interpersonal problems. Responses are scored on a 0–2 scale with “2” representing the severe form of a depressive symptom and “0” representing the absence of that symptom. The cutoff value is 19. Cronbach’s α was 0.86–0.95 (25).

### Outcomes

The primary outcomes were remission of depression in youths and the association between remission of depressive symptoms and perceived interparental conflicts in youths and interparental communication. Depressive symptoms were assessed before and after treatment according to CDI. The perceived interparental conflicts were examined using CPIC before and after treatment. Interparental communication satisfaction was assessed through indicators of constructive communication, such as constructive communication patterns and cooperating communication strategies, and indicators of destructive communication including demand/withdraw communication patterns, mutual avoidance communication patterns, and communication strategies, such as verbal aggression, physical aggression, child involvement, stonewalling, and avoidance–capitulation, before and after treatment according to the CPS and CPQ.

The secondary outcomes were the improvement of interparental communication satisfaction, reduction of perceived interparental conflicts in youths, and enhancement of marital satisfaction and fitness before and after treatment according to the CPS, CPQ, CPIC, CPI, QMI, and DAS. The raters were both psychiatrists (GBL and QY) and blinded to the participants’ treatment conditions.

### Intervention

The present study adopted a 5-week online SCGT for parents with depressed youths. The treatment protocol was based on family systems theory with the characteristics of short duration and intensive interventions. It contained one preparation session (approximately 2 hours), five intervention sessions (2 hours each session), and four discussion sessions (approximately 1 hour each session). Each participant underwent 16 treatment hours in total. Intervention and discussion session was conducted once a week; moreover, there was no discussion session in the fifth week. The experimental material was presented through word sheets. The detailed interview outlines are presented in the **Supplementary Material**. Two instances of non-attendance were considered dropping out of the study. In the current study, one participant dropped out of the study due to COVID-19 infection. The remaining subjects participated in all the sessions.

Interventions were provided by a qualified clinical psychologist (WYL) and a graduate student in clinical psychology (MTJ) who were trained in systemic family therapy and systemic couple therapy. They noted treatment records after each treatment and scored whether key treatment points were followed. A senior psychiatrist and psychologist (TDH) supervised the interventions.

### Statistical analyses

In the present study, the Shapiro–Wilk test was employed to verify the normality of data because our study was a small sample trial. A paired-sample *t*-test was used for data of normal distribution, while the Wilcoxon signed-rank test was applied to data of non-normal distribution to compare differences in depressive symptoms, perceived interparental conflicts, perceived interparental communication in youths, and the interparental communication satisfaction, marital satisfaction, and fitness in parents before and after interventions. The effect sizes were estimated using Cohen’s d; in the current study, the effect sizes of 0.2–0.3 were small, 0.5 medium, and ≥0.8 large (26). Subgroup analyses for gender and stress levels were performed.

The associations between depression, adolescents’ perceived changes, and interparental changes were further examined to show whether adolescents’ perceived changes and interparental changes affected the change in depression level. Spearman’s correlation analysis was used because some data had a non-normal distribution. LJJ and YJL performed the data analysis. All analyses were performed in SPSS 22.0, and *p* < 0.05 (two-sided) was considered significant.

## Findings

**Figure 1:**
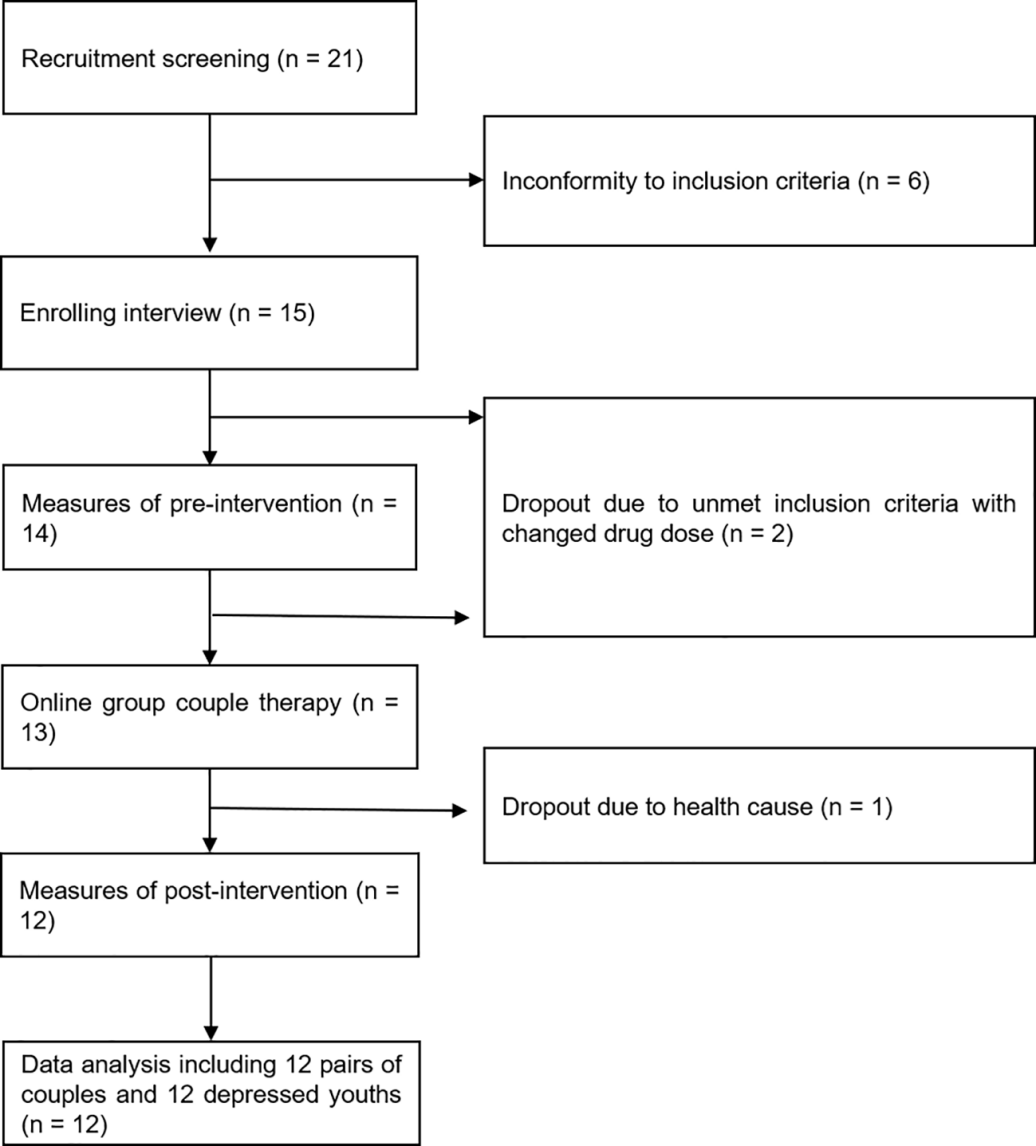
The flowchart of the systemic couple group therapy for families with depressed youths.

The flowchart of the present study is presented in **Figure 1**. We included data from 12 couples and 12 depressed youths ultimately. The mean age of parents was 45.13 years. The education of 19 (79.2%) participants was bachelor or above. The mean marriage year was 17.7, and 70.8% (17) of adult participants had severe marital stress (DAS ≤ 80).

The mean age of adolescents was 13 years. The youths were an even mix of genders, and the mean age of male youths was 12 years, while the mean age of female youths was 14 years (**Table 1**).

**Table 1:** Demographic characteristics of enrolled participants.

Parental data	
Sample sizes (n)	24
Age (years)	
30–39	4 (16.7%)
40–49	17 (70.8%)
50–59	3 (12.5%)
Years of marriage	11–25.2
Education	
Bachelor or above	19 (79.2%)
Below bachelor	5 (20.8%)
Family income (yuan/month)	5,000–50,000
10,000–30,000	16 (66.7%)
Number of children	
Only one	11 (91.7%)
Two	1 (8.3%)
DAS score (M ± SD)	62.7 ± 25.5
Adolescent data	
Age (years)	7–17
Gender	
Male	6 (50%)
Female	6 (50%)

DAS, dyadic adjustment scale.

### Children’s depression did not improve significantly post-intervention

The Wilcoxon signed-rank test was used to assess CDI differences before and after the intervention and found that there was a downward trend; however, no significant differences were seen in the depression level of children in the present study. Furthermore, Cohen’s d was 0.33, a small effect size (*p* = 0.258).

### Parental communication quality markedly improved post-intervention

The paired-sample *t*-test indicated that the global communication quality and satisfaction significantly improved, with a large effect size (Cohen’s d = 1.11, *p <* 0.001). Constructive communication patterns significantly improved after intervention as well, and the effect size was large (Cohen’s d = 0.85, *p <* 0.001). Other communication patterns, such as demand/withdraw (Cohen’s d = 0.69, *p* = 0.003) and mutual avoidance (Cohen’s d = 0.52, *p* = 0.018), and communication strategies, like verbal aggression (Cohen’s d = 0.55, *p* = 0.012), stonewalling (Cohen’s d = 0.71, *p* = 0.002), avoidance–capitulation (Cohen’s d = 0.46, *p* = 0.036), and child involvement (Cohen’s d = 0.79, *p* = 0.001), also reduced (**Table 2**).

**Table 2:** Comparisons of communication and marriage quality pre- and post-intervention.

Paired-samples *t*-test		Pre-intervention (M ± SD)	Post-intervention (M ± SD)	t	Cohen’s d
Communication satisfaction	Communication satisfaction	50.13 ± 11.24	61.46 ± 11.19	4.97^***^	1.11
Communication patterns	Constructive	−4.38 ± 9.97	6.42 ± 10.66	4.17^***^	0.85
	Demand/withdraw	36.67 ± 10.02	27.83 ± 8.57	−3.37^**^	0.69
	Mutual avoidance	15.17 ± 5.79	11.58 ± 4.67	−2.56^*^	0.52
Communication strategies	Verbal aggression	22.71 ± 5.79	18.17 ± 6.54	−2.72^*^	0.55
	Stonewalling	18.04 ± 3.38	14.58 ± 4.40	−3.47^**^	0.71
	Avoidance–capitulation	23.63 ± 4.44	21.83 ± 3.31	−2.23^*^	0.46
	Child involvement	14.50 ± 3.49	10.25 ± 4.13	−3.85^**^	0.79
Marriage quality	Subjective satisfaction	20.88 ± 11.16	32.13 ± 10.44	5.07^***^	1.03
Wilcoxon signed-rank test		Pre-intervention (M ± SD)	Post-intervention (M ± SD)	Z	
Communication strategies	Physical aggression	10.50 ± 3.40	8.71 ± 2.66	−2.58^*^	
	Cooperation	23.88 ± 5.02	26.42 ± 4.12	−2.18^*^	
Communication frequency	Communication frequency	3.00 ± 0.72	3.75 ± 0.79	−3.14^**^	
Marriage quality	Marital fitness	62.67 ± 25.51	86.08 ± 26.20	−3.49^***^	

^*^p < 0.05; ^**^p < 0.01; ^***^p < 0.001.

The Wilcoxon signed-rank test indicated that communication frequency (Z = 3.14, *p* = 0.002) and cooperation strategy (Z = 2.18, *p* = 0.029) increased and physical aggression reduced (Z = −2.58, *p* = 0.010) (**Table 2**).

We further evaluated whether gender and DAS level impact the communication improvements, indicating that no significant differences were seen in gender and DAS level (DAS < 80 vs. DAS < 95 and DAS ≥ 80).

### Parental marital quality markedly improved post-intervention

The findings of the paired-sample *t*-test demonstrated that significant improvements in subjective marital satisfaction occurred, with a large effect size (Cohen’s d = 1.03, *p <* 0.001). The findings of the Wilcoxon signed-rank test for DAS indicated that marriage adjustment significantly improved (Z = 3.49, p < 0.001) (**Table 2**). The average marital stress decreased from a severe level (62.67 ± 25.51) to a moderate or mild level (86.08 ± 26.20) through a 5-week intervention. The proportion of severe marital stress declined from 70.8% before intervention to 29.2% after intervention.

In addition, there were no significant differences for gender in both subjective and objective marital quality. However, compared with the mild/moderate stress level, better improvement was seen in the severe stress level for subjective marital satisfaction (13.00 ± 12.22 vs. 7.00 ± 5.07) and the DAS (29.76 ± 31.81 vs. 8.00 ± 9.78).

### Children’s perceived parental conflicts decreased post-intervention

The findings of the paired-sample *t*-test demonstrated that marital conflict frequencies (*p* = 0.043) and intensity (*p* = 0.028) decreased, while marital conflict solutions (*p* = 0.025) and coping efficacy (*p* = 0.038) enhanced. The effect sizes were medium with 0.66 for conflict frequency, 0.73 for conflict intensity, 0.75 for conflict solutions, and 0.68 for coping efficacy. Also, the effect size was medium for perceived threat (Cohen’s d = 0.57), although it was not significant (*p* = 0.072) (**Table 3**). There were no significant gender differences in terms of the perceived conflict measures.

**Table 3:** Perceived interparental conflicts of children pre- and post-intervention (paired-samples *t*-test).

	Pre-intervention (M ± SD)	Post-intervention (M ± SD)	t	Cohen’s d
Conflict frequency	16.83 ± 2.44	13.83 ± 4.63	−2.29^*^	0.66
Conflict intensity	17.08 ± 3.06	14.33 ± 4.46	−2.53^*^	0.73
Conflict resolution	15.33 ± 3.42	12.25 ± 4.45	−2.59^*^	0.75
Coping efficacy	18.67 ± 3.75	15.67 ± 4.25	2.36^*^	0.68
Perceived threat	17.00 ± 5.12	14.50 ± 4.56	1.99	0.57
Self-attribution	11.92 ± 3.92	11.83 ± 3.22	0.14	0.04
Conflict content	10.67 ± 2.87	10.67 ± 3.14	0.00	0.00

^*^p < 0.05.

### Correlations between interparental and children’s perceived changes

Parent values were averaged when considering correlations with adolescent data because we found that there were no gender differences in terms of measured variables.

For interparental relationships, subjective marital satisfaction had positive correlations with communication satisfaction (r = 0.70, *p <* 0.001) and constructive communication patterns (r = 0.58, *p* = 0.003) but negative correlations with demand/withdraw patterns (r = −0.62, *p* = 0.001), mutual avoidance patterns (r = −0.47, *p* = 0.022), verbal and physical aggression (r = −0.52, *p* = 0.009, and r = −0.47, *p* = 0.021, respectively), stonewalling (r = −0.55, *p* = 0.006), and child involvement (r = −0.48, *p* = 0.019). The DAS (marital fitness) positively correlated with communication satisfaction (r = 0.74, *p <* 0.001) and constructive communication patterns (r = 0.51, *p* = 0.012) but negatively correlated with mutual avoidance patterns (r = −0.41, *p* = 0.049).

For adolescents, depression level was significantly negatively correlated with interparental communication satisfaction (r = −0.52, *p* = 0.009) and interparental constructive communication patterns (r = −0.41, *p* = 0.044) and positively correlated with interparental mutual avoidance (r = 0.49, *p* = 0.016), marital satisfaction (r = 0.62, *p* = 0.001), and marital fitness (r = 0.70, *p <* 0.001). Perceived interparental conflict level positively correlated to interparental mutual avoidance (r = 0.46, *p* = 0.025), marital satisfaction (r = 0.43, *p* = 0.036), marital fitness (r = 0.67, *p <* 0.001), and depression level (r = 0.66, *p <* 0.001) (**Table 4**).

**Table 4:** Correlations between interparental and children’s perceived changes.

	1	2	3	4	5	6	7	8	9	10	11	12	13	14	15
1. Communication satisfaction	–														
2. Constructive communication	0.45^*^	–													
3. Demand/withdraw communication	−0.50^*^	−0.48^*^	–												
4. Mutual avoidance communication	−0.34	−0.64^**^	0.42^*^	–											
5. Verbal aggression	−0.30	−0.66^**^	0.59^**^	0.39	–										
6. Physical aggression	−0.42^*^	−0.46^*^	0.40	0.05	0.74^**^	–									
7. Cooperation	0.51^*^	0.35	−0.34	−0.28	−0.42^*^	−0.31	–								
8. Stonewalling	−0.31	−0.58^**^	0.31	0.31	0.75^**^	0.74^**^	−0.16	–							
9. Avoidance capitulation	−0.14	−0.11	0.17	0.12	0.35	0.12	−0.01	0.25	–						
10. Child involvement	−0.27	−0.52^**^	0.57^**^	0.30	0.89^**^	0.66^**^	−0.39	0.62^**^	0.47^*^	–					
11. Communication frequency	0.44^*^	0.21	−0.39	−0.40	−0.06	−0.11	0.24	−0.08	−0.09	−0.13	–				
12. Marital satisfaction	0.70^**^	0.58^**^	−0.62^**^	−0.47^*^	−0.52^**^	−0.47^**^	0.37	−0.55^**^	−0.01	−0.48^*^	0.19	–			
13. Marital fitness	0.74^**^	0.51^*^	−0.37	−0.41^*^	−0.33	−0.40	0.36	−0.37	−0.05	−0.32	0.11	0.73^**^	–		
14. Depression of children	−0.52^**^	−0.41^*^	0.66	0.49^*^	0.24	0.33	−0.18	0.34	0.29	0.10	−0.06	0.62^**^	0.70^**^	–	
15. Children’s perceived interparental conflict	−0.29	−0.27	0.21	0.46^*^	0.27	0.28	0.05	0.32	0.05	0.26	−0.07	0.43^*^	0.67^**^	0.66^**^	–

^*^p < 0.05; ^**^p < 0.01.

## Discussion

We designed the couple group therapy based on family systems theory to test the short efficacy of the intervention on families with depressed children and its effect on child depression changes. We found that SCGT could effectively improve interparental relationships and enhance marital satisfaction and fitness. It had a small effect size in improving the depressive symptoms of youths. There were positive relationships between interparental communication, marital satisfaction, and perceived interparental conflict and depression. Our study offers a potential way for families with depressed children who are unwilling to seek help.

There was a downtrend in depression scores after intervention without significance. One reason is limited to the small sample, and another is due to the short treatment duration. It could also be not enough to improve depression in children only through interparental intervention, and further studies are needed. However, the decrease in children’s depression scores was positively correlated with the increase in parents’ communication satisfaction, constructive communication patterns, the decrease in parents’ mutual avoidance patterns, and the reduction of perceived interparental conflicts. This provides an important direction for future clinical work; that is, parents need interventions to help them improve communication, which is likely to have a benefit on depressive symptoms for their children, especially for those unwilling to seek treatment. Children’s awareness of parental conflicts may be a mediating factor between parental conflict and childhood depression. In addition, a measure for suicidal ideation and the association between suicidal ideation and marital conflicts is needed in the future. Since suicidal ideation or suicide and self-harm risk among adolescents is a big step above depression, it is vital to reveal the role of marital conflicts in youths’ suicidal ideation and suicide and self-harm risk.

Rathgeber et al. found that the majority of participants in couple therapy were under mild/moderate marital stress; however, participants of the present study at baseline were under severe marital stress, indicating that SCGT could be an effective approach even for those couples with severe marital difficulties (27). Compared to couples with mild/moderate difficulties, greater improvements were seen in participants with severe marital difficulties. It indicated that in terms of marital quality, the current intervention was particularly effective for the severely affected population, which may be due to the unique therapeutic factor of group therapy, which is universality, which can lead group members to feel less alone or unique in their problems or level of misery (28). The spillover hypothesis from family systems theory suggests that the positivity or negativity experienced in the interparental relationship may transfer to the parent–child relationship (29). Emphasizing the importance of positive changes experienced in the interparental relationship on children would increase parents’ sense of efficacy, further transferring high stress to intensive hope and power.

Consistent with previous data, we also found that the more constructive the communication, the better the marital quality, and the more destructive the communication, the worse the marital quality (30). There were large effect sizes of communication satisfaction and constructive communication patterns. Medium effect sizes were seen in the demand/withdraw communication patterns, mutual avoidance patterns, verbal aggression, stonewalling, and child involvement, while there was a small effect size in the avoidance–capitulation strategy. It suggests that SCGT could enhance interparental communications. Both subjective marital satisfaction and fitness were positively correlated with communication satisfaction and constructive communication patterns and negatively correlated with mutual avoidance communication patterns in the present study. However, multiple destructive patterns and strategies affected subjective marital satisfaction, such as demand/withdraw patterns, verbal and physical aggression, stonewalling, and child involvement, which is in accordance with previous findings that 90.4% of the variance in marital satisfaction can be accounted for by couples’ communication (21). This is because destructive marital conflict involves more negative conflict resolution tactics, including aggressive and threatening behavior, arguing frequently, and leaving issues unresolved (11).

Perceived interparental conflict intensity and frequency declined, which is consistent with the decrease in interparental verbal and physical aggression and child involvement. In addition, the perceived increase in solving ability for marital conflict is consistent with the improvement of interparental constructive communication patterns and cooperative strategy, and the decline of demand/withdraw patterns, mutual avoidance patterns, and stonewalling strategy. Significant improvement in coping efficacy when facing interparental conflicts may be directly related to a decrease in the intensity and frequency of interparental conflicts and destructive communication patterns or an indirect benign internalization process. When parents are able to resolve conflicts that arise, the distress of children significantly reduces, and even if the conflict is not completely resolved, the distress reduces as well, which is positively correlated with the degree of resolution (31). It indicated that even if conflicts between parents persist, as long as it can help parents better handle conflicts, it can have a positive impact on the family and children. This highlights the importance of incorporating parent–child relationships when attempting to understand the underlying pathways between marital conflict and child functioning. However, for children, self-attribution and conflict contents related to themselves did not significantly change. A possible explanation is the limited efficacy of the intervention on children’s spontaneous emotional response and attribution style in a short time. Changes (aggressive behavior) in subsystems take a longer time to occur (29).

## Limitations

There were several limitations in our study. First, the study was a single group, and only within-group changes were evaluated. This greatly affected the accuracy of the results and increased the possibility of biased results caused by factors that threaten internal and external validity. Second, a small sample size in the present study may result in inflation of Type 1 errors, and we interpreted findings with caution. A larger sample size (such as more than 400) trial should be conducted in the future using a structural equation modeling to multi-level modeling for the adolescent/parent structure of data to further offer a reliable conclusion. Third, the present study is not a randomized controlled trial (RCT), and the non-random selection of samples rendered further causal inferences of results. RCTs with large samples are needed to validate the efficacy of SCGT further in the future. Fourth, it would be better if there were qualitative data in the present study. Fifth, the treatment duration was limited, which may have hindered significant changes in some indicators requiring a longer time to occur, such as changes in children’s depressive symptoms, automated emotions, and attribution styles. Sixth, it is unclear how the impact of SCGT on participating families changes over time, as well as whether it will continue and for how long. Future research should extend the treatment cycle appropriately to bring about more significant therapeutic effects and be conducted with multiple follow-ups like 4 weeks, 6 months, or 1 year after the end of the intervention.

## Conclusion and clinical relevance

The SCGT could significantly enhance interparental communication satisfaction, constructive communication patterns and strategies, communication frequencies, marital satisfaction, and fitness while reducing destructive communication patterns and strategies. Depressed youths also perceived less interparental conflict intensity, frequency and difficulty in conflict resolution, and a higher sense of self-efficacy. Helping parents improve communication and marital quality may affect depression symptoms for children in their families. The SCGT offers a possibility for the treatment of families with depressed children who are unwilling to seek treatment: spillover positive changes in the parental subsystem into the children subsystem through family systems dynamics and processes.

## Data availability statement

The raw data supporting the conclusions of this article will be made available by the authors, without undue reservation.

## Ethics statement

The studies involving humans were approved by ethics committee/institutional review board of Peking University Sixth Hospital. The studies were conducted in accordance with the local legislation and institutional requirements. Written informed consent for participation in this study was provided by the participants’ legal guardians/next of kin. All data collected were anonymous, and the study was conducted following the ethical principles of the World Medical Association Declaration of Helsinki of 1975, as revised in 2008.

## Author contributions

T-JM: Investigation, Writing – original draft. YQ: Supervision, Writing – review & editing. Y-LW: Investigation, Writing – review & editing. B-LG: Methodology, Writing – review & editing. J-JL: Writing – review & editing. J-LY: Funding acquisition, Writing – review & editing. D-HT: Supervision, Writing – review & editing.

## References

[1] Polanczyk GV Salum GA Sugaya LS Caye A Rohde LA. Annual research review: a meta-analysis of the worldwide prevalence of mental disorders in children and adolescents. J Child Psychol psychiatry Allied disciplines. (2015) 56:345–65. doi: 10.1111/jcpp.12381 25649325

[2] WHO. Adolescents: health risks and solutions. Geneva: World Health Organisation (2018). Available at: http://www.who.int/en/news-room/fact-sheets/detail/adolescents-health-risks-and-solutions.

[3] McLeod GFH Horwood LJ Fergusson DM. Adolescent depression, adult mental health and psychosocial outcomes at 30 and 35 years. psychol Med. (2016) 46:1401–12. doi: 10.1017/S0033291715002950 26818194

[4] Mojtabai R Olfson M Han B. National trends in the prevalence and treatment of depression in adolescents and young adults. Pediatrics. (2016) 138(6):e20161878. doi: 10.1542/peds.2016-1878 27940701 PMC5127071

[5] Galimberti C Bosi MF Volontè M Giordano F Dell'Osso B Viganò CA. Duration of untreated illness and depression severity are associated with cognitive impairment in mood disorders. Int J Psychiatry Clin Pract. (2020) 24:227–35. doi: 10.1080/13651501.2020.1757116 32338553

[6] Yap MB Pilkington PD Ryan SM Jorm AF. Parental factors associated with depression and anxiety in young people: a systematic review and meta-analysis. J Affect Disord. (2014) 156:8–23. doi: 10.1016/j.jad.2013.11.007 24308895

[7] NICE Evidence Reviews Collection. Psychological interventions for the treatment of depression: depression in children and young people, 2019 Evidence review A. London: National Institute for Health and Care Excellence (NICE) (2019). 32479041

[8] Goodyer IM Reynolds S Barrett B Byford S Dubicka B Hill J. Cognitive behavioural therapy and short-term psychoanalytical psychotherapy versus a brief psychosocial intervention in adolescents with unipolar major depressive disorder (IMPACT): a multicentre, pragmatic, observer-blind, randomised controlled superiority trial. Lancet Psychiatry. (2017) 4:109–19. doi: 10.1016/s2215-0366(16)30378-9 PMC5285447 27914903

[9] Hammen C Rudolph K Weisz J Rao U Burge D. The context of depression in clinic-referred youth: neglected areas in treatment. J Am Acad Child Adolesc Psychiatry. (1999) 38:64–71. doi: 10.1097/00004583-199901000-00021 9893418

[10] Brock RL Kochanska G. Interparental conflict, children's security with parents, and long-term risk of internalizing problems: a longitudinal study from ages 2 to 10. Dev Psychopathol. (2016) 28:45–54. doi: 10.1017/s0954579415000279 25797703 PMC4580501

[11] Hosokawa R Katsura T. Exposure to marital conflict: gender differences in internalizing and externalizing problems among children. PloS One. (2019) 14:e0222021. doi: 10.1371/journal.pone.0222021 31513615 PMC6742467

[12] Kitzmann KM. Effects of marital conflict on subsequent triadic family interactions and parenting. Dev Psychol. (2000) 36:3–13. doi: 10.1037//0012-1649.36.1.3 10645740

[13] Sturge-Apple ML Davies PT Cummings EM. Impact of hostility and withdrawal in interparental conflict on parental emotional unavailability and children's adjustment difficulties. Child Dev. (2006) 77:1623–41. doi: 10.1111/j.1467-8624.2006.00963.x 17107450

[14] Rao U Chen LA. Characteristics, correlates, and outcomes of childhood and adolescent depressive disorders. Dialogues Clin Neurosci. (2009) 11:45–62. doi: 10.31887/DCNS.2009.11.1/urao 19432387 PMC2766280

[15] Hammen C. Stress generation in depression: reflections on origins, research, and future directions. J Clin Psychol. (2006) 62:1065–82. doi: 10.1002/jclp.20293 16810666

[16] Spanier GB. Measuring dyadic adjustment: new scales for assessing the quality of marriage and similar dyads. J marriage Family. (1976) 38:15–28. doi: 10.2307/350547

[17] Crane DR Middleton KC Bean RA. Establishing criterion scores for the Kansas Marital Satisfaction Scale and the Revised Dyadic Adjustment Scale. Am J Family Ther. (2000) 28:53–60. doi: 10.1080/019261800261815

[18] Norton R. Measuring marital quality: a critical look at the dependent variable. J Marriage Family. (1993) 45:141–51. doi: 10.2307/351302

[19] Kerig PK. Assessing the links between interparental conflict and child adjustment: the conflicts and problem-solving scales. J Family Psychol. (1996) 10:454–73. doi: 10.1037//0893-3200.10.4.454

[20] Pickover AM Dodson TS Tran HN Lipinski AJ Beck JG. Factor structure of the Communication Patterns Questionnaire in violence-exposed women. J interpersonal violence. (2021) 36:9352–70. doi: 10.1177/0886260519867147 31387450

[21] Vazhappilly JJ Reyes MES. Couples' communication as a predictor of marital satisfaction among selected Filipino couples. psychol Stud. (2016) 61:1–6. doi: 10.1007/s12646-016-0375-5

[22] Furukawa R Driessnack M. Testing the committee approach to translating measures across cultures: translating primary communication inventory from English to Japanese. Nurs Health Sci. (2016) 18:450–56. doi: 10.1111/nhs.12291 27325230

[23] Grych JH Seid M Fincham FD. Assessing marital conflict from the child's perspective: the Children's Perception of Interparental Conflict Scale. Child Dev. (1992) 63:558–72. doi: 10.1111/j.1467-8624.1992.tb01646.x 1600822

[24] Nikolas M Klump KL Burt SA. Etiological contributions to the covariation between Children's Perceptions of Inter-parental Conflict and child behavioral problems. J Abnormal Child Psychol. (2013) 41:239–51. doi: 10.1007/s10802-012-9679-7 PMC3543475 22996155

[25] Saylor CF Finch AJ Jr. Baskin CH Saylor CB Darnell G Furey W. Children's Depression Inventory: investigation of procedures and correlates. J Am Acad Child Psychiatry. (1984) 23:626–8. doi: 10.1016/s0002-7138(09)60357-5 6481037

[26] Larner AJ. Effect size (Cohen's d) of cognitive screening instruments examined in pragmatic diagnostic accuracy studies. Dementia geriatric Cogn Disord extra. (2014) 4:236–41. doi: 10.1159/000363735 PMC4132220 25177332

[27] Rathgeber M Bürkner PC Schiller EM Holling H. The efficacy of emotionally focused couples therapy and behavioral couples therapy: a meta-analysis. J marital Family Ther. (2019) 45:447–63. doi: 10.1111/jmft.12336 29781200

[28] Hoge MA McLoughlin KA. Group psychotherapy in acute treatment settings: theory and technique. Hosp Community Psychiatry. (1991) 42:153–8. doi: 10.1176/ps.42.2.153 1997364

[29] Cox MJ Paley B. Understanding families as systems. Curr Dir psychol Sci. (2003) 12(5):193–6. doi: 10.1111/1467-8721.01259

[30] Stinson MA Bermúdez JM Gale J Lewis D Meyer AS Templeton GB. Marital satisfaction, conflict resolution styles, and religious attendance among Latino couples. Family J. (2017) 25(3):215–23. doi: 10.1177/1066480717710645

[31] Goeke-Morey MC Cummings EM Papp LM. Children and marital conflict resolution: implications for emotional security and adjustment. J Family Psychol. (2007) 21:744–53. doi: 10.1037/0893-3200.21.4.744 18179346

