# Supplementary material for: The effect of systematic couple group therapy on families with depressed juveniles: a pilot trial
Source: Front Psychiatry. 2024 May 28;15:1283519. doi: 10.3389/fpsyt.2024.1283519 (PMC11165141; doi:10.3389/fpsyt.2024.1283519)
Supplement: Supplementary file 1 [file Table_1.docx]

Supplementary material

Table 1. Sessions and outlines of group systematic couple therapy for family with depressed children or adolescents

| Session | Outlines and contents |
| --- | --- |
| Inclusion interview | - Outline：Introduction，Q&A, preliminary information collection and evaluation; motivation stimulation |
|  | - Introduction of group settings, treatment schedule, contents and expected results of each treatment; lay stress on confidentiality, the importance of participation and homework requirements, etc. |
|  | - Answer other questions and concerns about group therapy to prepare for entry into the group - Collection of basic family information; Preliminary assessment; Motivation discussion; Preliminary discussion on family communication patterns |
|  |  |
| Session 1  Session 2  Session 3  Session 4  Session 5 | - Main content: circular causation theory - Introduction of group members (members introduce themselves and share their personal experiences that led them to the group) - Explain the group structure and meeting arrangements; description of group rules and suggestions for participation - Motivation stimulation; talking about the importance for parents of improving the quality of communication and the relationship - Introduction of the overall goals of group therapy - Psychological education: Introduction of circular causation thinking among the interparental interaction, especially during conflicts - Discussion: circular causation thinking and interpreting examples in real life situations - Homework assignment: Observe what situations in the couple's life can be considered in circular causation way of thinking; Experience it and take notes - Main content: Education of good communication - Homework review and discussion - Discussion: What do your ideal communication look like? - Psychological education: effective communication and ineffective communication - Discussion: What good experience of effective communication do you and your partner have in your marriage? - Homework assignment: red ledger to record separately each other's progress and advantages in daily communication - Main content: How to deal with divergence of views and express different opinions - Homework review and discussion - Psychological education: ways to negotiate issues and express different opinions (observation and statement; experience feelings; reflect on needs; express requests) - Live practice and discussion - Assign homework: Practice the negotiation method in life and record the specific process - Main content: listening to each other - Homework review, consolidation and discussion - Psychological education: How to listen and give feedback in the face of conflict or difficult situations - Live practice and discussion - Assign homework: Practice listening to each other and giving feedbacks in real life, and record the specific process - Main contents: consolidation and making future plans - Homework review, consolidation and discussion - Review the main themes and contents of previous four group activities to consolidate the effect - Changes discussion and results sharing: the most impressive part, one’s own changes, and the practice plan for the next month - Psychological education: caring for oneself - Conclusions; end of group activities |
